# Complexity and Uniqueness of the Aromatic Profile of Smoked and Unsmoked Herreño Cheese

**DOI:** 10.3390/molecules19067937

**Published:** 2014-06-12

**Authors:** Gemma Palencia, Maria Luisa Ibargoitia, Maria Fresno, Patricia Sopelana, Maria Dolores Guillén

**Affiliations:** 1 Food Technology, Faculty of Pharmacy, Lascaray Research Center, University of the Basque Country (UPV/EHU), Paseo de la Universidad n° 7, 01006 Vitoria, Spain; E-Mails: gemmaclara.palencia@ehu.es (G.P.); marialuisa.ibargoitia@ehu.es (M.L.I.); patricia.sopelana@ehu.es (P.S.); 2 Animal Production Unit, Canary Agronomic Science Institute, 38200 La Laguna, Tenerife, Spain; E-Mail: mfresno@icia.es

**Keywords:** smoked and unsmoked Herreño cheese, headspace components, solid phase microextraction, gas chromatography-mass spectrometry

## Abstract

In this work, the volatile fraction of unsmoked and smoked Herreño cheese, a type of soft cheese from the Canary Islands, has been characterized for the first time. In order to evaluate if the position in the smokehouse could influence the volatile profile of the smoked variety, cheeses smoked at two different heights were studied. The volatile components were extracted by Solid Phase Microextraction using a divinylbenzene/carboxen/polydimethylsiloxane fiber, followed by Gas Chromatography/Mass Spectrometry. In total, 228 components were detected. The most numerous groups of components in the unsmoked Herreño cheese were hydrocarbons, followed by terpenes and sesquiterpenes, whereas acids and ketones were the most abundant. It is worth noticing the high number of aldehydes and ketones, and the low number of alcohols and esters in this cheese in relation to others, as well as the presence of some specific unsaturated hydrocarbons, terpenes, sesquiterpenes and nitrogenated derivatives. The smoking process enriches the volatile profile of Herreño cheese with ketones and diketones, methyl esters, aliphatic and aromatic aldehydes, hydrocarbons, terpenes, nitrogenated compounds, and especially with ethers and phenolic derivatives. Among these, methylindanones or certain terpenes like α-terpinolene, have not been detected previously in other types of smoked cheese. Lastly, the results obtained suggest a slightly higher smoking degree in the cheeses smoked at a greater height.

## 1. Introdution

Herreño cheese comes from El Hierro, the smallest of the Canary Islands. El Hierro Island has been a territory traditionally linked to livestock raising, in which herds were mixed, so its traditional cheese is manufactured with milk from different animal species. Concretely, the Herreño cheese is manufactured with a mixture of pasteurized milk from goat, cow and sheep (the three of Canary breed). This type of cheese occupies an important position in the Canary Islands market for smoked soft cheeses; however, its volatile fraction, known to be important in cheese flavour, has never been studied.

In spite of the great number of studies carried out, the knowledge about volatile cheese components has advanced very slowly. It is well known that the volatile component profile of a cheese sample can appear to be different depending on the technique and methodology used for its study. Some techniques are only able to extract volatile components of low molecular weight, and others are not able to extract components of certain functionality; moreover, there are significant differences in relation to the repeatability of the techniques used [1,2,3]. This makes difficult to compare the results obtained in different studies and, consequently, to compare the volatile composition of different types of cheese accurately. It should also be mentioned that, among the various techniques used for the study of cheese volatiles, Solid Phase Microextraction (SPME) coupled to Gas Chromatography/Mass Spectrometry (GC/MS) has been increasingly used since its development in the nineties, due to the advantages that this technique offers regarding simplicity and speed of analysis [4,5]. However, it is worth noticing that the type of fibre used can also lead to differences in the results obtained by SPME, since the different phases exhibit different affinity for the various cheese components, depending on their molecular weight or polarity, as example [4]. This technique has been used by some authors to determine absolute concentrations of free fatty acids in ewe cheese [6] and of a more varied group of components in white surface mould cheeses [7]. Nevertheless, it is difficult to achieve an exact and reliable quantification when a high number of volatile compounds, belonging to different chemical families, is studied.

In this work, the study of the volatile fraction of unsmoked and smoked Herreño cheese has been carried out by means of SPME followed by GC/MS for the subsequent study of the extracted compounds. The purpose of this work is, on the one hand, to characterize the components of the headspace of unsmoked and smoked Herreño cheese, and, on the other hand, to establish the influence of the smoking process on the volatile profile of this type of cheese. This will contribute to define the aromatic profile of this type of cheese and maybe to find specific markers that could be useful to distinguish Herreño cheese from other cheese varieties.

Furthermore, the influence of the cheese position in the smokehouse on the volatile components of the cheese is also subject of attention in this study. Finally, this study will add to knowledge about the volatile fraction of smoked cheeses. This has, to date, been studied very little.

## 2. Results and Discussion

### 2.1. Characterization of the Unsmoked and of the Smoked Herreño Cheese

**Figure 1:**
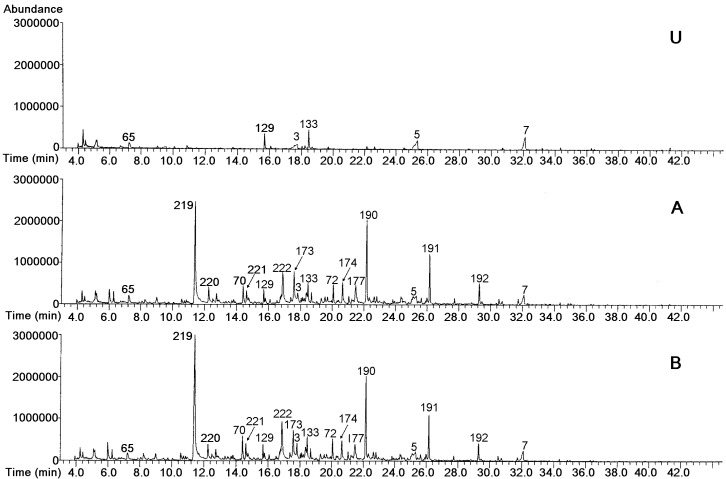
Total ion current chromatogram of the components of the headspace of the unsmoked cheese (**U**), and of the cheeses smoked at positions **A** and **B**.

**Figure 2:**
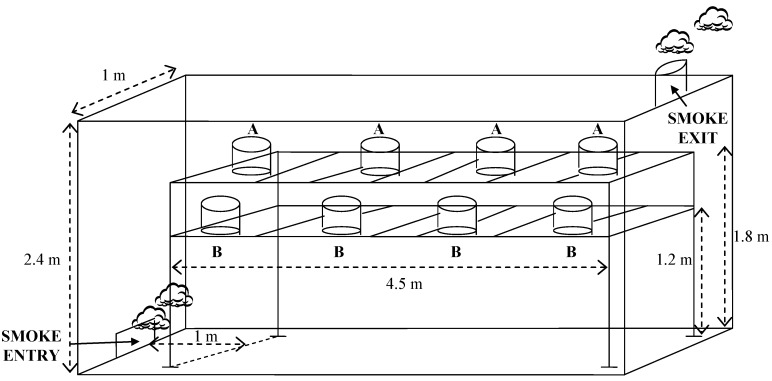
Scheme of the position of the A and B Herreño cheeses in the smokehouse.

Figure 1 shows the total ion chromatograms corresponding to the headspace of the exterior region of the unsmoked cheese (U) and of those smoked at both positions A and B, indicated in Figure 2. The numbers of the components agree with those in Table 1, Table 2, Table 3, Table 4 and Table 5. This figure shows that the volatile profiles of the smoked cheeses (A and B) were very similar, but clearly different from that of the unsmoked one; this latter was much poorer in volatile compounds.

The compounds identified in the studied Herreño cheeses, grouped in families according to their chemical nature, together with their average area counts, divided by 10^5^, are given in Table 1, Table 2, Table 3, Table 4 and Table 5. Asterisked compounds were identified by comparison with commercial standard compounds. It is worth noticing the high number (228) of components detected, which show a broad range of molecular weights and volatilities.

Although the number of components in the unsmoked cheese (120) was much lower than in the smoked ones (221), in both cheese varieties the most numerous group was that of hydrocarbons.

Furthermore, in the unsmoked cheese, the groups of compounds having the highest area counts are acids and ketones, followed by alcohols and terpenes and sesquiterpenes; however, in the smoked cheeses, the groups with the highest area counts are by far ethers and phenolic derivatives, followed by ketones and diketones.

#### 2.1.1. Acids, Alcohols, Esters and Lactones

The compounds found of these groups are shown in Table 1. Although acids are common cheese components [8,9,10], it could be said that Herreño cheese is particularly rich in these components, since they are very numerous and have a high number of area counts. As it is usual, those of even number of carbon atoms, especially hexanoic and octanoic, are in much higher abundance than those of uneven number of carbon atoms [11], both in the unsmoked and in the smoked cheeses. 

**Table 1:** Acids, alcohols, esters and lactones detected in the headspace of the exterior region of unsmoked (U) and smoked (A and B) Herreño cheeses studied, together with their molecular weight, the base peak of their mass spectra (Bp), their abundance, expressed as average area counts of their mass spectra base peak divided by 10^5^, and standard deviation ^a^.

Nº	Compounds (Molecular Weight)	Bp	U	A	B
*Acids*					
	*Linear*				
1	Acetic acid (60) *	43	18.09 ± 8.32 ^a^	32.97 ± 7.13 ^a^	31.60 ± 4.75 ^a^
2	Butanoic acid (88) *	60	25.99 ± 2.82 ^b^	14.11 ± 2.56 ^a^	15.83 ± 1.22 ^a^
3	Hexanoic acid (caproic acid) (116) *	60	56.21 ± 4.80 ^b^	35.93 ± 3.88 ^a^	41.75 ± 5.36 ^a^
4	Heptanoic acid (130) *	60	1.24 ± 0.17 ^a^	1.77 ± 0.32 ^a^	1.70 ± 0.05 ^a^
5	Octanoic acid (caprilic acid) (144) *	60	43.27 ± 0.93 ^a^	35.94 ± 4.44 ^a^	40.85 ± 4.08 ^a^
6	Nonanoic acid (158) *	60	1.30 ± 0.14 ^a^	1.09 ± 0.10 ^a^	1.03 ± 0.21 ^a^
*Acids*					
7	Decanoic acid (capric acid) (172) *	60	28.55 ± 3.34 ^b^	17.31 ± 2.67 ^a^	21.27 ± 3.99 ^ab^
8	Dodecanoic acid (lauric acid) (200) *	73	0.97 ± 0.07 ^b^	0.55 ± 0.22 ^a^	0.50 ± 0.01 ^a^
9	Hexadecanoic acid (palmitic acid) (256) *	73	-	0.09 ± 0.03 ^a^	0.16 ± 0.05 ^a^
	*Branched*				
10	2-Ethylhexanoic acid (144)	88	0.52 ± 0.00 ^b^	0.35 ± 0.01 ^a^	0.31 ± 0.04 ^a^
	*Aromatic*				
11	Benzoic acid (122)	105	10.94 ± 0.12 ^b^	4.44 ± 1.12 ^a^	5.03 ± 1.34 ^a^
*Alcohols*					
	*Aliphatic*				
12	Ethanol (46) *	45	79.86 ± 0.24 ^b^	43.52 ± 6.46 ^a^	45.12 ± 1.64 ^a^
13	3-Methyl-1-butanol (88) (or isomer)	55	2.74 ± 0.55	-	-
14	1-Pentanol (amilol) (86)	42	1.16 ± 0.00	-	-
	*Aromatic*				
15	Benzenemethanol (108) *		-	6.64 ± 0.76 ^a^	7.08 ± 1.21 ^a^
*Esters*					
	*Aliphatic*				
16	Methyl acetate (74)	43	-	8.48 ± 0.26 ^a^	10.86 ± 0.79 ^b^
17	Methyl propenoate (86)	55	-	1.88 ± 0.27 ^a^	2.91 ± 0.61 ^a^
18	Methyl hydroxyacetate (90)	58	-	2.83 ± 0.41 ^a^	1.95 ± 0.33 ^a^
19	Methyl-2-butenoate (100)	69	-	3.88 ± 0.02 ^a^	3.98 ± 0.84 ^a^
20	Ethyl decanoate (200) *	88	0.17 ± 0.01 ^a^	0.18 ± 0.01 ^a^	0.21 ± 0.03 ^a^
21	1-Methylethyl hexadecanoate (298)	43	-	0.27 ± 0.08 ^a^	0.25 ± 0.24 ^a^
	*Aromatic*				
24	Methyl 4-methylbenzoate (150)	119	-	1.49 ± 0.34 ^a^	1.48 ± 0.11 ^a^
*Lactones*					
	*γ-Lactones*				
25	2(3H)-Dihydrofuranone (γ-butyrolactone) (86)	42	-	5.52 ± 0.64 ^a^	5.13 ± 0.67 ^a^
26	2(5H)-Furanone (γ-crotonolactone) (84) *	55	-	13.67 ± 1.93^a^	11.48 ± 1.12 ^a^
27	5-Methyl-2(5H)furanone (98) *	55	-	3.80 ± 0.32 ^a^	3.74 ± 0.39 ^a^
28	3-Methyl-2(5H)furanone (98) *	41	-	8.47 ± 0.66 ^a^	8.45 ± 0.55 ^a^
29	5-Methyl-5-ethyldihydro-2(3H)-furanone (128)	99	-	2.88 ± 0.42 ^a^	2.53 ± 0.11 ^a^
30	γ-Dodecalactone (198) *	85	-	0.31 ± 0.02 ^a^	0.34 ± 0.06 ^a^
	*δ-Lactones*				
31	3,6-Dimethyl-2H-pyran-2-one (124)	68	-	2.06 ± 0.30 ^a^	1.83 ± 0.18 ^a^
32	3-Hydroxy-2-methyl-4H-pyran-4-one (maltol) (126) *	126	-	3.30 ± 0.67 ^a^	2.60 ± 0.46 ^a^
33	δ-Octalactone (148)	99	0.62 ± 0.04 ^a^	0.67 ± 0.07 ^a^	0.83 ± 0.10 ^a^
34	δ-Decalactone (170) *	99	1.56 ± 0.09 ^a^	1.62 ± 0.13 ^a^	1.86 ± 0.06 ^a^
35	δ-Dodecalactone (198) *	99	0.49 ± 0.03 ^a^	0.50 ± 0.09 ^a^	0.52 ± 0.02 ^a^

^a^ Different letters in the same row indicate significant statistical differences (Tukey’s Test, *p* < 0.05). * Asterisked compounds were identified by means of standard compounds.

**Table 2:** Aldehydes, ketones and diketones detected in the headspace of the exterior region of unsmoked (U) and smoked (A and B) Herreño cheeses studied, together with their molecular weight, the base peak of their mass spectra (Bp), their abundance, expressed as average area counts of their mass spectra base peak divided by 10^5^, and standard deviation ^a^.

Nº	Compounds (Molecular Weight)	Bp	U	A	B
*Aldehydes*					
	*Aliphatic saturated*				
36	Pentanal (86)	44	0.69 ± 0.07 ^a^	0.48 ± 0.04 ^a^	0.54 ± 0.13 ^a^
37	Hexanal (100) *	56	2.33 ± 0.18 ^b^	1.08 ± 0.14 ^a^	1.09 ± 0.13 ^a^
38	Heptanal (114)	70	0.17 ± 0.01 ^a^	0.26 ± 0.06 ^a^	0.24 ± 0.03 ^a^
39	Nonanal (142) *	57	2.51 ± 0.25 ^a^	2.50 ± 0.29 ^a^	3.18 ± 0.19 ^a^
40	Decanal (156)	43	0.33 ± 0.04	-	-
41	Dodecanal (miristaldehyde) (184)	57	0.29 ± 0.01 ^a^	0.28 ± 0.01 ^a^	0.28 ± 0.03 ^a^
42	Tridecanal (268)	57	-	0.10 ± 0.01 ^a^	0.14 ± 0.06 ^a^
43	Tetradecanal (212)	57	0.22 ± 0.01 ^a^	0.24 ± 0.04 ^a^	0.24 ± 0.05 ^a^
	*Aliphatic unsaturated*				
44	2-Butenal (crotonaldehyde) (70)	70	0.41 ± 0.04 ^a^	57.18 ± 1.83 ^b^	64.60 ± 16.91 ^b^
45	2-Methyl-2-butenal (or isomer) (84)	84	-	1.84 ± 0.22 ^a^	1.81 ± 0.29 ^a^
46	2-Pentenal (84)	55	0.56 ± 0.00 ^a^	3.95 ± 0.22 ^b^	4.53 ± 1.15 ^b^
47	2-Nonenal (140)	41	0.20 ± 0.02	-	-
	*Aromatic*				
48	Benzaldehyde (106) *	105	2.04 ± 0.30 ^a^	29.33 ± 3.31 ^b^	33.04 ± 5.13 ^b^
49	Benzeneacetaldehyde (phenylacetaldehyde) (120) *	91	1.13 ± 0.03 ^a^	2.71 ± 0.54 ^b^	3.07 ± 0.39 ^b^
50	2-Hydroxy-benzaldehyde (salicylaldehyde) (122)	122	-	9.11 ± 1.63 ^a^	10.75 ± 2.00 ^a^
51	Methylbenzaldehyde (120)	119	-	9.28 ± 1.42 ^a^	10.53 ± 1.72 ^a^
52	Methylbenzaldehyde (120)	119	-	3.77 ± 0.76 ^a^	4.12 ± 0.43 ^a^
53	3-Hydroxy-4-methylbenzaldehyde (136)	136	-	3.41 ± 0.64 ^a^	3.41 ± 0.55 ^a^
54	4-Hydroxy-3-methoxybenzaldehyde (vanillin) (152) *	152	-	0.92 ± 0.09 ^a^	0.82 ± 0.21 ^a^
*Ketones and diketones*					
*Ketones*					
	*Aliphatic*				
55	2-Propanone (58) *	43	27.89 ± 0.38 ^a^	22.92 ± 6.68 ^a^	25.49 ± 4.74 ^a^
56	2-Butanone (72) *	43	7.28 ± 1.59 ^a^	4.05 ± 0.64 ^a^	4.91 ± 1.34 ^a^
57	2-Pentanone (86) *	43	3.09 ± 0.07 ^a^	6.29 ± 0.52 ^b^	6.93 ± 1.42 ^b^
58	4-Methyl-3-pentanone (100)	57	0.42 ± 0.02 ^a^	2.08 ± 0.10 ^b^	2.40 ± 0.66 ^b^
59	3-Penten-2-one (84)	69	-	8.97 ± 0.90 ^a^	8.86 ± 1.42 ^a^
60	2-Heptanone (114) *	43	5.95 ± 0.34 ^a^	9.38 ± 0.10 ^b^	9.33 ± 1.35 ^b^
61	3,3-Dimethyl-2-butanone (100)	57	0.30 ± 0.02 ^a^	22.48 ± 1.30 ^b^	23.14 ± 3.97 ^b^
62	2-Nonanone (142) *	58	3.37± 0.05 ^a^	7.76 ± 0.61 ^b^	7.77 ± 1.37 ^b^
63	2-Undecanone (170)	58	0.33 ± 0.03 ^a^	0.46 ± 0.03 ^b^	0.50 ± 0.06 ^b^
	*Oxygenated aliphatic*				
64	1-Hydroxy-2-propanone (74)	43	2.94 ± 0.28 ^a^	51.81 ± 7.23 ^b^	48.59 ± 2.39 ^b^
65	3-Hydroxy-2-butanone (acetoin) (88) *	45	48.84± 8.81 ^a^	39.80 ± 16.25 ^a^	32.64 ± 0.24 ^a^
66	1-(Acetyloxy)-2-propanone (116) *	43	0.83 ± 0.05 ^a^	59.52 ± 1.33 ^b^	59.15 ± 9.49 ^b^
	*Cyclic*				
67	Cyclopentanone (84) *^b^	55	0.97 ± 0.02 ^a^	4.96 ± 0.84 ^b^	6.57 ± 1.00 ^b^
68	Cyclohexanone (98) *	55	0.28 ± 0.00 ^a^	3.69 ± 0.57 ^b^	4.66 ± 0.67 ^b^
69	2-Methylcyclopentanone (98)	69	0.14 ± 0.00 ^a^	1.58 ± 0.09 ^b^	1.47 ± 0.25 ^b^
70	3-Methyl-2-cyclopenten-1-one (96) *	96	1.98 ± 0.01 ^a^	43.49 ± 7.12 ^b^	47.70 ± 6.44 ^b^
71	2-Cyclohexen-1-one (96)	68	-	4.90 ± 0.82 ^a^	5.31 ± 1.29 ^a^
72	Dimethylcyclopent-2-en-1-one (110)	67	0.54 ± 0.01 ^a^	6.84 ± 1.28 ^b^	8.12 ± 0.93 ^b^
73	Dimethylcyclopent-2-en-1-one (110)	95	-	7.57 ± 0.15 ^a^	6.78 ± 0.53 ^a^
74	2-Methyl-2-cyclopenten-1-one (96) *	96	0.72 ± 0.02 ^a^	3.43 ± 0.20 ^b^	3.37 ± 0.55 ^b^
75	Dimethylcyclopent-2-en-1-one (110)	95	0.45 ± 0.04 ^a^	11.94 ± 1.26 ^b^	14.64 ± 2.71 ^b^
76	3-Methyl-2-cyclohexen-1-one (110)	82	-	3.85 ± 0.45 ^a^	4.33 ± 0.67 ^a^
77	Trimethyl-2-cyclopenten-1-one (124)	109	-	9.84 ± 0.86 ^a^	9.36 ± 1.01 ^a^
78	Dimethylcyclopent-2-en-1-one (110)	67	1.22 ± 0.03 ^a^	40.61 ± 3.46 ^b^	41.45 ± 7.27 ^b^
79	Trimethyl-2-cyclopenten-1-one (124)	109	0.47 ± 0.01 ^a^	15.75 ± 1.46 ^b^	16.69 ± 2.65 ^b^
	*Aromatic*				
80	1-(Methylphenyl)-ethanone (134) (or isomer)	119	-	4.58 ± 0.46 ^a^	4.23 ± 0.63 ^a^
81	2,3-Dihydro-1H-inden-1-one (indanone) (132)	132	-	6.91 ± 1.25 ^a^	5.34 ± 0.90 ^a^
82	3-Methyl-1-indanone (146)	131	-	2.33 ± 0.27 ^a^	1.77 ± 0.28 ^a^
83	7-Methyl-1-indanone (146)	117	-	1.28 ± 0.16 ^a^	0.98 ± 0.16 ^a^
84	4-Methyl-1-indanone (146)	117	-	0.62 ± 0.12 ^a^	0.50 ± 0.12 ^a^
85	1-(2,6-Dihydroxy-4-methoxyphenyl)-ethanone (acetovanillone) (182) *	167	-	1.69 ± 0.33 ^a^	1.17 ± 0.21 ^a^
*Diketones*					
	*Linear*				
86	2,3-Butanedione (diacetyl) (86) *	43	7.70 ± 4.19 ^a^	6.19 ± 0.75 ^a^	7.91 ± 0.94 ^a^
87	2,3-Pentanedione (100)	43	-	4.53 ± 0.27 ^a^	5.13 ± 1.04 ^a^
88	2,5-Hexanedione (114) *	43	-	6.16 ± 0.36 ^a^	6.32 ± 1.36 ^a^
	*Cyclic*				
89	Cyclopent-2-en-1,4-dione (96)	96	-	20.43 ± 3.14 ^a^	23.86 ± 3.27 ^a^
90	3-Methyl-1,2-cyclopentanedione (cyclotene) (112) *	112	-	17.12 ± 3.42 ^a^	12.98 ± 2.36 ^a^
91	4-Ethyl-cyclopentan-1,2-dione (126)	97	-	9.18 ± 0.52 ^a^	9.26 ± 1.64 ^a^

^a^ Different letters in the same row indicate significant statistical differences (Tukey’s Test, *p* < 0.05). * Asterisked compounds were identified by means of standard compounds.

The concentration of acids differs in the unsmoked and in the smoked cheeses. Thus, except acetic acid, most of them are in higher concentration in the former than in the latter; this indicates that the smoking process enriches the cheese headspace in acetic acid, while causing a decrease in most of the other ones.

Concerning alcohols, it must be pointed out that Herreño cheese is poor in this type of components, above all the smoked one. Thus, among the three primary aliphatic alcohols detected in the unsmoked cheese, only ethanol is present in the smoked cheeses, and in significantly lower abundance than in the former; this evidences the influence of the smoking process on the occurrence of alcohols in this cheese. On the other hand, it should be noticed the presence in the smoked cheeses of benzenemethanol, a well known smoke component [12].

**Table 3:** Hydrocarbons, terpenes and sesquiterpenes detected in the headspace of the exterior region of unsmoked (U) and smoked (A and B) Herreño cheeses studied, together with their molecular weight, the base peak of their mass spectra (Bp), their abundance, expressed as average area counts of their mass spectra base peak divided by 10^5^, and standard deviation ^a^.

Nº	Compounds (Molecular Weight)	Bp	U	A	B
*Hydrocarbons*					
	*Aliphatic*				
92	4-Methyl-2-pentene (84)	69	-	8.97 ± 0.90 ^a^	8.86 ± 1.42 ^a^
93	2-Heptene (98)	57	1.93 ± 0.34 ^a^	2.02 ± 0.34 ^a^	2.59 ± 0.34 ^a^
94	Nonane (128) *	43	0.40 ± 0.02 ^a^	0.59 ± 0.06 ^b^	0.79 ± 0.07 ^b^
95	*cis*-3,7-Dimethyl-2-octene (140) (or isomer)	70	1.42 ± 0.12 ^a^	1.14 ± 0.20 ^a^	1.16 ± 0.26 ^a^
96	Branched hydrocarbon	57	11.57 ± 2.82 ^a^	14.52 ± 3.75 ^a^	12.07 ± 2.59 ^a^
97	Linear hydrocarbon	57	1.38 ± 0.29 ^a^	2.95 ± 0.16 ^b^	2.56 ± 0.06 ^b^
98	Linear hydrocarbon	57	2.70 ± 0.15 ^a^	3.87 ± 0.34 ^b^	3.34 ± 0.32 ^b^
99	Linear hydrocarbon	57	0.80 ± 0.11 ^a^	1.01 ± 0.11 ^a^	1.20 ± 0.20 ^a^
100	Undecane (156)	43	0.43 ± 0.08 ^a^	0.68 ± 0.02 ^ab^	0.76 ± 0.17 ^b^
101	Branched hydrocarbon	57	0.47 ± 0.07 ^a^	0.68 ± 0.08 ^b^	0.72 ± 0.09 ^b^
102	Dodecane (170) *	57	0.52 ± 0.07 ^a^	0.72 ± 0.08 ^b^	0.76 ± 0.12 ^b^
103	Tridecane (184)	43	0.28 ± 0.01 ^a^	0.52 ± 0.02 ^b^	0.54 ± 0.06 ^b^
104	Tetradecane (198) *	57	2.88 ± 0.02 ^b^	1.25 ± 0.10 ^a^	1.34 ± 0.08 ^a^
105	Pentadecane (212) *	57	0.32 ± 0.07 ^ab^	0.26 ± 0.01 ^a^	0.36 ± 0.01 ^b^
106	Hexadecane (226) *	57	0.20 ± 0.01 ^a^	0.23 ± 0.03 ^a^	0.25 ± 0.04 ^a^
107	3,7,11,15-Tetramethyl-2-hexadecene (phytene) (280)	70	0.23 ± 0.00 ^a^	0.20 ± 0.12 ^a^	0.21 ± 0.07 ^a^
	*Monoaromatic*				
108	Benzene (78)	78	0.50 ± 0.05 ^a^	6.79 ± 1.63 ^b^	8.72 ± 0.87 ^b^
109	Toluene (92) *	91	12.52 ± 2.03 ^a^	19.75 ± 3.62 ^b^	20.45 ± 4.67 ^b^
110	Dimethylbenzene (106)	91	0.95 ± 0.16 ^a^	4.03 ± 0.66 ^b^	4.33 ± 0.37 ^b^
111	Dimethylbenzene (106)	91	2.89 ± 0.07 ^a^	8.23 ± 1.37 ^b^	10.78 ± 2.09 ^b^
112	Styrene (104) *	104	1.75 ± 0.16 ^a^	13.22 ± 3.67 ^b^	12.25 ± 2.06 ^b^
113	Methylmethylethylbenzene (isomer) (134)	119	2.27 ± 0.03 ^a^	2.20 ± 0.36 ^a^	2.60 ± 0.06 ^a^
	*Polyaromatic*				
114	1H-Indene (116)	130	0.35 ± 0.08 ^a^	9.13 ± 1.60 ^b^	11.42 ± 0.63 ^b^
115	1-Methyl-1*H*-indene (130)	130	-	2.74 ± 0.69 ^a^	2.80 ± 0.41 ^a^
116	2-Methyl-1*H*-indene (130)	130	-	1.60 ± 0.20 ^a^	1.80 ± 0.11 ^a^
117	Azulene (128)	128	0.09 ± 0.05 ^a^	2.49 ± 0.69 ^b^	2.47 ± 0.24 ^b^
118	Naphtalene (128) *	128	1.84 ± 0.56 ^a^	37.49 ± 7.91 ^b^	36.87 ± 2.67 ^b^
119	Decahydronaphthalene (138)	138	-	1.06 ± 0.19 ^a^	0.86 ± 0.15 ^a^
120	2-Methylnaphthalene (142) *	142	0.17 ± 0.06 ^a^	3.09 ± 0.11 ^b^	3.00 ± 0.29 ^b^
121	1-Methylnaphthalene (142) *	142	0.10 ± 0.06 ^a^	2.57 ± 0.45 ^b^	2.09 ± 0.15 ^b^
122	1,1'-Biphenyl (154)	154	-	1.07 ± 0.15 ^a^	0.88 ± 0.06 ^a^
123	Dimethylnaphthalene (156)	156	-	0.33 ± 0.05 ^a^	0.28 ± 0.03 ^a^
124	Dimethylnaphthalene (156)	156	-	0.40 ± 0.05 ^b^	0.29 ± 0.01 ^a^
125	Dimethylnaphthalene (156)	156	-	0.13 ± 0.02 ^a^	0.10 ± 0.01 ^a^
126	Acenaphthylene (152) *	152	-	0.99 ± 0.08 ^b^	0.73 ± 0.06 ^a^
*Terpenes*					
127	δ-3-Carene (136)	93	1.23 ± 0.13 ^a^	0.89 ± 0.20 ^a^	0.86 ± 0.09 ^a^
128	α-Thujene (136)	93	0.73 ± 0.07 ^a^	0.69 ± 0.18 ^a^	0.70 ± 0.14 ^a^
129	α-Pinene (136) *	93	34.91 ± 1.79 ^a^	25.68 ± 5.49 ^a^	24.71 ± 2.58 ^a^
130	Camphene (136) *	93	1.65 ± 0.00 ^a^	1.20 ± 0.20 ^a^	1.09 ± 0.21 ^a^
131	β-Terpinene (136) *	93	0.51 ± 0.06 ^a^	0.59 ± 0.08 ^a^	0.70 ± 0.05 ^a^
132	β-Pinene (136) *	93	2.39 ± 0.06 ^a^	4.86 ± 0.68 ^b^	4.79 ± 0.64 ^b^
133	Camphane (138)	95	29.33 ± 0.71 ^b^	18.94 ± 3.08 ^a^	20.81 ± 1.28 ^a^
134	α-Phellandrene (136) *	93	3.78 ± 0.14 ^a^	2.00 ± 0.20 ^a^	2.41 ± 0.18 ^a^
135	Menth-1-ene (138)	138	0.17 ± 0.00 ^a^	0.98 ± 0.23 ^b^	0.89 ± 0.03 ^b^
136	*trans*-Carane (138) or *trans*-*p*-Menth-1-ene (138)	95	0.41 ± 0.01	-	-
137	α-Limonene (136) *	68	2.68 ± 0.12 ^a^	5.78 ± 0.58 ^b^	6.71 ± 1.62 ^b^
138	α-Fenchene (136)	93	0.12 ± 0.02 ^a^	0.73 ± 0.17 ^b^	0.76 ± 0.05 ^b^
139	Alloocimene (136)	121	-	0.87 ± 0.38 ^a^	1.10 ± 0.17 ^a^
140	α-Terpinolene (136)	136	-	1.73 ± 0.24 ^a^	1.70 ± 0.41 ^a^
141	α-Terpinene (136) *	136	-	0.26 ± 0.04 ^b^	0.15 ± 0.02 ^a^
142	*β*-Camphor (152) *^b^	152	-	0.32 ± 0.05 ^a^	0.46 ± 0.11 ^a^
*Sesquiterpenes*					
143	α-Copaene (or isomer) (204)	161	0.44 ± 0.01 ^b^	0.32 ± 0.02 ^a^	0.39 ± 0.05 ^ab^
144	Cyperene (or isomer) (204)	204	0.21 ± 0.00 ^a^	0.28 ± 0.02 ^ab^	0.32 ± 0.06^ b^
145	α-Gurjunene (or isomer) (204)	161	0.06 ± 0.00 ^a^	0.11 ± 0.00 ^b^	0.13 ± 0.03 ^b^
146	Junipene (or isomer) (204)	161	0.09 ± 0.01 ^a^	0.11 ± 0.02 ^a^	0.11 ± 0.04 ^a^
147	*trans*-Caryophyllene (204) *	93	1.08 ± 0.02 ^a^	1.06 ± 0.05 ^a^	1.06 ± 0.37 ^a^
148	Alloaromadendrene (or isomer) (204)	161	0.07 ± 0.00 ^a^	0.12 ± 0.01 ^b^	0.14 ± 0.03 ^b^
149	α-Humulene (or isomer) (204)	93	0.21 ± 0.00 ^a^	0.21 ± 0.01 ^a^	0.22 ± 0.00 ^a^
150	Widdrene (or isomer) (204)	119	0.16 ± 0.05 ^a^	0.17 ± 0.02 ^a^	0.20 ± 0.01 ^a^
151	Calarene (or isomer) (204)	161	0.09 ± 0.02 ^a^	0.13 ± 0.02 ^a^	0.15 ± 0.03 ^a^
152	β-Cadinene (or isomer) (204)	204	0.05 ± 0.00 ^a^	0.04 ± 0.00 ^a^	0.05 ± 0.01 ^a^
153	Valencene (or isomer) (204)	161	0.07 ± 0.00 ^a^	0.08 ± 0.01 ^a^	0.09 ± 0.02 ^a^

^a^ Different letters in the same row indicate significant statistical differences (Tukey’s Test, *p* < 0.05). * Asterisked compounds were identified by means of standard compounds.

It is also noteworthy that the headspace of unsmoked Herreño cheese is almost free of esters, except for a few lactones, which are cyclic esters; this contrasts with that observed in the smoked cheeses, where esters are quite numerous. The esters detected in the smoked Herreño cheese are mainly methyl esters, above all aliphatic but also aromatic, being methyl acetate and methyl benzoate the most abundant within each group, respectively.

The presence of these compounds almost exclusively in the smoked cheeses indicates that their origin is in the smoking process, either as components coming from the smoke [13,14] or as components resulting from reactions that can take place during this process between alcohols and acids present in cheese. This latter hypothesis would be in agreement with the lower number and concentrations of alcohols, and also with the decrease in the concentrations of most acids in the headspace of the smoked cheeses in relation to the unsmoked one. As an example, it can be seen in Table 1 that, in the smoked cheeses, the increase in the concentration of methylbenzoate is in line with the decrease in benzoic acid.

**Table 4:** Nitrogenated derivatives detected in the headspace of the exterior region of unsmoked (U) and smoked (A and B) Herreño cheeses studied, together with their molecular weight, the base peak of their mass spectra (Bp), their abundance, expressed as average area counts of their mass spectra base peak divided by 10^5^, and standard deviation ^a^.

Nº	Compounds (Molecular Weight)	Bp	U	A	B
*Nitrogenated derivatives*					
	*Nitrile derivatives*				
154	Benzonitrile (103)	103	1.66 ± 0.25 ^a^	53.89 ± 5.98 ^b^	63.61 ± 9.01 ^b^
155	3-Methylbenzonitrile (117)	117	0.04 ± 0.02 ^a^	1.50 ± 0.25 ^b^	1.73 ± 0.20 ^b^
156	Benzeneacetonitrile (117)	117	-	2.40 ± 0.38 ^a^	2.34 ± 0.64 ^a^
157	Benzenepropanenitrile (131)	91	-	2.14 ± 0.38 ^a^	1.76 ± 0.25 ^a^
	*Pyridine and pyrazine derivatives and others*				
158	Pyrazine (80)	80	0.60 ± 0.04 ^a^	6.79 ± 0.58 ^b^	7.92 ± 1.51 ^b^
159	Pyridine (79) *	79	2.16 ± 0.06 ^a^	29.84 ± 0.43 ^b^	34.88 ± 5.80 ^b^
160	4-Methylpyridine (93)	93	0.71 ± 0.20 ^a^	11.39 ± 1.54 ^b^	13.61 ± 1.78 ^b^
161	Methylpyrazine (94)	94	0.73 ± 0.03 ^a^	12.97 ± 1.02 ^b^	15.13 ± 3.00 ^b^
162	2-Methylpyridine (93)	93	0.61 ± 0.05 ^a^	19.92 ± 2.50 ^b^	22.55 ± 3.31 ^b^
163	2,6-Dimethylpyridine (107) *	107	-	2.15 ± 0.36 ^a^	2.08 ± 0.31 ^a^
164	2-Ethylpyridine (107) *	106	0.19 ± 0.01 ^a^	4.40 ± 1.00 ^b^	5.64 ± 0.96 ^b^
165	2,4-Dimethylpyridine (107) *	107	-	6.12 ± 1.06 ^a^	7.76 ± 1.27 ^a^
166	3,4-Dimethylpyridine (107)	107	-	2.49 ± 0.36 ^a^	2.70 ± 0.32 ^a^
167	3-Ethylpyridine (107)	92	-	2.49 ± 0.45 ^a^	2.88 ± 0.42 ^a^
168	3-Methoxypyridine (109) *	109	-	15.14 ± 0.64 ^a^	15.81 ± 2.07 ^a^
169	2-Ethyl-6-methylpyridine (121)	120	-	1.86 ± 0.30 ^a^	1.86 ± 0.22 ^a^
170	5-Ethyl-2-methylpyridine (121) *	120	-	2.02 ± 0.24 ^a^	2.42 ± 0.48 ^a^
171	2-Methoxy-3-methylpyrazine (124)	124	-	7.05 ± 0.53 ^a^	6.58 ± 1.25 ^a^
172	β-Nicotyrine (158)	158	0.53 ± 0.11 ^a^	0.61 ± 0.12 ^a^	0.60 ± 0.01 ^a^

^a^ Different letters in the same row indicate significant statistical differences (Tukey’s Test, *p* < 0.05). * Asterisked compounds were identified by means of standard compounds.

Lactones, as commented above, are cyclic esters resulting from hydroxy acids and they can be divided into furanones and pyranones. Furanones (γ-lactones) are only present in the smoked cheeses. However, among the pyranones (δ-lactones), 3,6-dimethyl-2H-pyran-2-one and maltol are also smoke components, whereas δ-octalactone, δ-decalactone and δ-dodecalactone are present both in the smoked and in the unsmoked cheeses in similar concentrations. This seems to be in agreement with previous results since, in smoked Palmero cheese [12,15], these latter lactones were detected both in the exterior and in the interior of the cheeses in similar concentrations, pointing to another origin than smoke.

**Table 5:** Phenol derivatives and ethers detected in the headspace of the exterior region of unsmoked (U) and smoked (A and B) Herreño cheeses studied, together with their molecular weight, the base peak of their mass spectra (Bp), their abundance, expressed as average area counts of their mass spectra base peak divided by 10^5^, and standard deviation ^a^.

Nº	Compounds (Molecular Weight)	Bp	U	A	B
*Phenol derivatives*					
	*Phenol*				
173	Phenol (94) *	94	-	173.11 ± 18.44 ^a^	149.26 ± 21.62 ^a^
174	2-Methylphenol (108)	107	-	46.52 ± 7.71 ^a^	39.09 ± 6.76 ^a^
175	3-Methylphenol (108) * +	107	-	79.36 ±15.04 ^a^	64.64 ± 10.73 ^a^
	4-Methylphenol (108) *				
176	2,6-Dimethylphenol (122) *	122	-	13.47 ± 1.23 ^a^	12.22 ± 1.90 ^a^
177	2-Ethylphenol (122) * +	107	-	6.32 ± 0.90 ^a^	5.37 ± 0.79 ^a^
	2-Propylphenol (136) *				
178	2,4-Dimethylphenol (122) *	122	-	12.32 ± 2.13 ^a^	10.00 ± 2.05 ^a^
179	2,5-Dimethylphenol (122) *	122	-	11.95 ± 1.78 ^a^	9.88 ± 1.84 ^a^
180	Dimethylphenol (122)	122	-	6.51 ± 1.17 ^a^	5.53 ± 0.60 ^a^
181	Dimethylphenol (122)	122	-	12.64 ± 2.34 ^a^	13.09 ± 2.51 ^a^
182	Dimethylphenol (122)	122	-	3.73 ± 0.59 ^a^	2.98 ± 0.55 ^a^
183	2-(1-Methylethyl)-phenol (136)	121	-	2.28 ± 0.13 ^a^	1.97 ± 0.45 ^a^
184	2,4,6-Trimethylphenol (136) *	121	-	1.73 ± 0.19 ^a^	1.44 ± 0.27 ^a^
185	2,3,5-Trimethylphenol (136) *	121	-	1.80 ± 0.19 ^a^	1.49 ± 0.24 ^a^
186	Diethylphenol (150)	135	-	1.93 ± 0.23 ^a^	1.73 ± 0.17 ^a^
187	3,4,5-Trimethylphenol (136) *	121	-	1.41 ± 0.22 ^a^	1.25 ± 0.23 ^a^
188	2,6-Bis(1,1-dimethylethyl)-4-methylphenol (BHT) (220) *	205	-	0.29 ± 0.04 ^a^	0.25 ± 0.00 ^a^
189	2,4,5-Tri-*sec*-buthylphenol (262)	203	-	0.32 ± 0.02 ^a^	0.34 ± 0.06 ^a^
	*Methoxyphenol derivatives*				
190	2-Methoxyphenol (guaiacol) (124) *	109	-	250.50 ± 24.70 ^a^	231.94 ± 33.36 ^a^
191	2-Methoxy-4-methylphenol (4-methylguaiacol) (138) *	123	-	107.65 ± 8.76 ^a^	88.25 ± 13.92 ^a^
192	4-Ethyl- 2-methoxyphenol (4-ethylguaiacol) (152) *	137	-	70.36 ± 9.01 ^a^	55.65 ± 8.93 ^a^
193	4-Vinyl-2-methoxyphenol (4-vinylguaiacol) (150) *	135	-	11.53 ± 0.95 ^a^	9.76 ± 2.10 ^a^
194	4-(2-Propenyl)-2-methoxyphenol (eugenol) (164) *	164	-	4.00 ± 0.62 ^a^	3.25 ± 0.66 ^a^
195	2-Methoxy-4-propylphenol (4-propylguayacol) (166) *	137	-	5.86 ± 0.67 ^a^	4.66 ± 0.79 ^a^
196	4-(1-Propenyl)-2-methoxyphenol (isoeugenol isomer) (164) *	164	-	1.50 ± 0.25 ^a^	1.16 ± 0.30 ^a^
197	4-(1-Propenyl)-2-methoxyphenol (isoeugenol isomer) (164) *	164	-	3.06 ± 0.42 ^a^	2.15 ± 0.26 ^a^
	*Dimethoxyphenol derivatives*				
198	Dimethoxyphenol (154) (isomer)	154	-	0.54 ± 0.01 ^a^	0.52 ± 0.07 ^a^
199	2,6-Dimethoxyphenol (syringol) (154) *	154	-	11.18 ± 0.22 ^a^	11.31 ± 1.93 ^a^
*Ethers*					
	*Alkyl-aryl ethers*				
200	Methoxybenzene (108)	108	-	3.41 ± 0.38 ^a^	3.91 ± 0.49 ^a^
201	4-Methyl-1-methoxybenzene (122)	122	-	7.01 ± 2.03 ^a^	8.82 ± 1.37 ^a^
202	Ethoxy-benzene (122)	94	-	3.01 ± 0.76 ^a^	3.49 ± 0.60 ^a^
203	1,1-Dimethylethoxybenzene (150)	94	-	1.10 ± 0.08 ^a^	0.96 ± 0.12 ^a^
204	1,2-Dimethoxybenzene (veratrol) (138) *	123	-	5.39 ± 0.67 ^a^	5.42 ± 1.26 ^a^
205	1,4-Dimethoxybenzene (138) *	123	-	12.69 ± 0.73 ^a^	11.81 ± 0.96 ^a^
206	Dimethoxytoluene (152) (isomer)	152	0.13 ± 0.01 ^a^	12.71 ± 2.14 ^b^	11.49 ± 2.57 ^b^
207	3,4-Dimethoxytoluene (152) (or isomer)	152	0.01 ± 0.00 ^a^	3.01 ± 0.34 ^b^	2.62 ± 0.27 ^b^
208	5-Methyl-1,2,3-trimethoxybenzene (182)	182	-	1.14 ± 0.04 ^b^	0.85 ± 0.17 ^a^
209	Trimethoxybenzene (168) (isomer)	168	-	3.39 ± 0.39 ^a^	2.94 ± 0.05 ^a^
	*Furan derivatives*				
	*Benzofuran derivatives*				
210	Benzofuran (118)	118	1.52 ± 0.09 ^a^	30.72 ± 4.44 ^b^	36.01 ± 5.76 ^b^
211	Methyl-benzofuran (132) (isomer)	131	0.12 ± 0.06 ^a^	3.10 ± 0.66 ^b^	3.47 ± 0.50 ^b^
212	Methyl-benzofuran (132) (isomer)	131	0.35 ± 0.14 ^a^	8.51 ± 2.04 ^b^	9.42 ± 1.05 ^b^
213	Methyl-benzofuran (132) (isomer)	131	0.45 ± 0.17 ^a^	10.81 ± 2.11 ^b^	11.34 ± 1.86 ^b^
214	Dimethyl-benzofuran (146) (isomer)	146	0.09 ± 0.03 ^a^	2.18 ± 0.24 ^b^	2.52 ± 0.20 ^b^
215	Dimethyl-benzofuran (146) (isomer)	146	0.16 ± 0.05 ^a^	3.83 ± 0.95 ^b^	3.81 ± 0.17 ^b^
216	Methoxy-benzofuran (148)	148	-	1.25 ± 0.08 ^b^	1.06 ± 0.09 ^a^
	*Others*				
217	2-Methylfuran (82)	82	-	3.06 ± 0.15 ^a^	6.36 ± 1.36 ^b^
218	3-Furaldehyde (96)	95	-	3.35 ± 0.70 ^a^	3.71 ± 0.86 ^a^
219	2-Furancarboxaldehyde (96) *	96	-	698.19 ± 105.95 ^a^	725.90 ± 148.68 ^a^
220	2-Furanmethanol (98) *	98	0.50 ± 0.16 ^a^	47.23 ± 5.12 ^b^	43.03 ± 7.10 ^b^
221	1-2-(Furanyl)-ethanone (2-acetylfuran) (110) *	95	2.60 ± 0.09 ^a^	81.61 ± 8.70 ^b^	90.94 ± 12.94 ^b^
222	5-Methyl-2-furancarboxaldehyde (110) *	110	0.96 ± 0.10 ^a^	153.68 ± 20.11 ^b^	144.53 ± 35.56 ^b^
223	Methyl furancarboxylate (126)	95	-	38.37 ± 3.51 ^a^	41.74 ± 7.58 ^a^
224	1-(2-Furanyl)-1-propanone (124)	95	-	11.41 ± 0.50 ^a^	11.86 ± 1.92 ^a^
225	2-Butyltetrahydrofuran (128)	71	-	31.85 ± 2.25 ^a^	30.46 ± 4.81 ^a^
226	2-(2-Propenyl)-furan (108)	79	-	3.40 ± 0.49 ^a^	3.77 ± 0.83 ^a^
227	5-Hydroxymethyl-2-furancarboxaldehyde (126) *	97	-	9.10 ± 0.65 ^a^	9.96 ± 2.26 ^a^
228	2-(2-Furanylmethyl)-5-methylfuran (162)	162	-	0.92 ± 0.10 ^a^	0.91 ± 0.01 ^a^

^a^ Different letters in the same row indicate significant statistical differences (Tukey’s Test, *p* < 0.05). * Asterisked compounds were identified by means of standard compounds.

#### 2.1.2. Aldehydes, Ketones and Diketones

Aldehydes constitute a very important group of components in both unsmoked and smoked Herreño cheese (see Table 2). Some of these aldehydes are well known smoke components [13] and, consequently, either they are only present in the smoked cheeses or are in these latter in higher proportions. Others, however, have generally similar concentrations in both cheese varieties. In short, as a consequence of the smoking process, the Herreño cheese is enriched greatly with aldehydes coming from the smoke.

It can be observed in Table 2 that, among the aliphatic saturated aldehydes, the most numerous and abundant ones in the unsmoked cheese, nonanal and hexanal, are those with the highest area count numbers.

On the other hand, aliphatic unsaturated aldehydes and those aromatic ones are the most abundant in the smoked cheeses, being the area counts of benzaldehyde and, above all, of crotonaldehyde (2-butenal), the highest. Two aromatic aldehydes have also been found in the unsmoked cheese, although in significantly lower concentration than in the smoked variety, being benzaldehyde the most abundant.

The ketones group is very numerous and, as it can be observed in Table 2, it is one of the majority group of volatiles in area counts, both in the smoked and in the unsmoked cheeses, being acetoin, a common cheese component [16], the most abundant in the unsmoked one.

Within the wide group of ketones, most of the aliphatic and cyclic ones are present both in the unsmoked and in the smoked cheeses; among these, there are several methylketones such as 2-pentanone, 2-heptanone, 2-nonanone and 2-undecanone, which are well known cheese components [17].

With smoking, the presence of ketones is increased considerably. Thus, as can be observed in the smoked variety, the smoking process not only increases the concentration of aliphatic and cyclic ketones in relation to the unsmoked cheese, but also provides the Herreño cheese with several aromatic ketones; these are typical smoke components [13] and are absent in the unsmoked variety. Among all these, the highest abundances correspond to 3-methyl-2-cyclopenten-1-one, dimethyl- and some trimethyl-cyclopentenones.

Within the diketones group, only diacetyl (2,3-butanedione), which is a common cheese component [16,17], has been detected in the unsmoked and in the smoked cheeses in similar concentration. The rest of diketones are only present in the smoked cheeses, being those cyclic more abundant than the linear ones. 

#### 2.1.3. Hydrocarbons, Terpenes and Sesquiterpenes

The headspace of the Herreño cheese contains a very high number of hydrocarbons, both aliphatic and aromatic, as Table 3 shows. Among the aliphatic hydrocarbons, most of them are in quite similar abundances in the unsmoked and in the smoked cheeses, even though 4-methyl-2-pentene is only present in the smoked variety, with a high number of area counts.

Regarding the aromatic hydrocarbons, their higher number and concentration in the smoked variety indicate that most of these come from the smoking process. Thus, although all the monoaromatic and some of the polyaromatic hydrocarbons have also been detected in the unsmoked cheese, they are, in general, in significantly lower concentrations than in the smoked ones.

The high abundance of toluene in the unsmoked cheese is noteworthy; this could be related to environmental contamination, as some authors have suggested [18], or even to the metabolism of *β*-carotene in milk [19]. The ingestion of contaminated feed by the animals producers of the milk could also explain the presence of polyaromatic hydrocarbons in the unsmoked cheese [20].

The group of terpenes and sesquiterpenes, also shown in Table 3, is, together with hydrocarbons, one of the most numerous in the unsmoked Herreño cheese. Moreover, the great number of terpenes and sesquiterpenes present in the headspace of Herreño cheese, both unsmoked and smoked, could be considered a distinctive characteristic of this cheese. Many of these compounds are present in similar concentration in the unsmoked and in the smoked cheeses, being α-pinene and camphane those with the highest abundances, in both varieties. As it is known, these compounds are secondary metabolites of plants, especially of aromatic plants, and their presence in both unsmoked and smoked Herreño cheese indicates that probably they were present in the milk used for cheese elaboration, and their origin could be associated with the feed of the folk.

However, as Table 3 shows, the smoking process also has a noticeable influence on the amount of terpenes in Herreño cheese. It not only enriches significantly its volatile profile in certain terpenes such as β-pinene or α-limonene, but also provides this cheese with new terpenes, not detected in the unsmoked variety, such as alloocimene, α-terpinolene, α-terpinene and β-camphor.

#### 2.1.4. Nitrogenated Derivatives

Another standing feature of the volatile composition of Herreño cheese is its content in nitrogen derivatives, shown in Table 4. They include nitrile and, above all, pyridine and pyrazine derivatives. Some of these components are present in the unsmoked cheese, but both their number and their concentration increase considerably with the smoking process, especially those of pyridine derivatives.

Within this group of nitrogenated derivatives, the alkaloid β-nicotyrine is the only one whose abundance is almost the same in the smoked and in the unsmoked cheeses, so its origin would be probably different from smoke. This compound, whose presence in cheese has only been reported in Idiazabal [11], has been described as one of the alkaloids present in *Nicotiana paniculata* [21], a potential invading species that grows in the Canary Islands.

#### 2.1.5. Phenol Derivatives and Ethers

Phenolic derivatives, shown in Table 5, are typical smoke components [13,14] and in fact, they are absent in the unsmoked Herreño cheese. They account for a high proportion of the total of volatile components in smoked Herreño cheese, both in number and in area counts. They are constituted by phenol and its derivatives, which are the most numerous, methoxy- and dimethoxyphenol derivatives, being the most abundant guaiacol (2-methoxyphenol), phenol and their derivatives.

Ethers, also shown in Table 5, are well known smoke components [14], although a few of them are also present in the unsmoked cheese, in much lower concentrations than in the smoked ones; among these, 2-acetylfuran can be mentioned due to its abundance.

This group includes alkyl-aryl ethers and furan derivatives, being these latter the most abundant in the smoked cheese. It is worth noticing the high abundance of 2-furancarboxaldehyde, which is the compound with the highest abundance, not only within this group of compounds, but also among all the volatile compounds detected in the smoked Herreño cheese.

### 2.2. Comparison of the Unsmoked Herreño Cheese with Other Types of Goat Cheeses

It is not easy to compare the volatile profile of different types of cheese when the technique used for the determination of such components is not the same. Besides, both the treatment of the milk or the cheese ripening degree influence to a great extent the volatile profile of each cheese. Despite this, some similarities have been found between the unsmoked Herreño cheese, produced mainly from goat milk, and other types of goat cheeses.

Some of the main components of the unsmoked Herreño cheese have also been found to be important in the volatile fraction of other goat cheeses; this is the case of acids, which constitute the main chemical family in some of them [22,23]. Concretely, hexanoic acid, the most abundant in this Herreño cheese (see Table 1), was also the predominant acid in other types of goat cheese such as the Spanish Ibores [22], the Turkish Gokceada cheese [23] or the Maltese goat cheese [24]. Moreover, acetic and butanoic acids, also quite abundant in the unsmoked Herreño cheese, were present at high concentrations in Ibores [22] and in Gokceada goat cheeses [23], being acetic acid the most abundant in Xinotyri [25] and Teleme goat cheeses [26].

On the other hand, ketones, which constitute another important group of volatile compounds in the unsmoked Herreño cheese, are among the major volatile components detected in Teleme goat cheese [26]. Among these, some methyl ketones such as 2-heptanone and 2-nonanone are present in different types of goat cheese [27,28,29]. It has also been found that acetoin, the most abundant ketone in the unsmoked Herreño cheese, was also among the most abundant in other goat cheeses [23,30,31]. Anyway, it should be noticed that the number of ketones detected in this Herreño cheese is higher than in other goat cheeses.

As commented above, one outstanding characteristic of this Herreño cheese is the low number of alcohols, which contrasts with the high abundance of alcohols in other goat milk cheeses [22,25,32]. According to the observations of Bontinis *et al.* [25], this difference could be due to the use of pasteurized milk for Herreño cheese production; in fact, in Teleme goat cheese [26], made also with pasteurized milk, alcohols were very scarce too. However, it is worth noticing that, irrespective of the alcohols abundance, both in raw and in pasteurized goat cheeses, ethanol is one of the most abundant [23,25,26], and this is also observed in Herreño cheese (see Table 1). The other two aliphatic alcohols present in the unsmoked Herreño cheese, 3-methyl-1-butanol and 1-pentanol, have also been found in other types of goat cheeses, above all the first one [23,27,31].

Esters also constitute another peculiar feature of unsmoked Herreño cheese since, in contrast with other goat cheeses [22,23,25], they are practically absent in Herreño cheese.

Although different aldehydes have been found in the headspace of other types of unsmoked goat cheese [23,32], it could be said that the unsmoked Herreño cheese is richer in aldehydes than other goat cheeses.

Something similar could be said of hydrocarbons since, whereas in Herreño cheese this group of components is the most numerous (see Table 3), only a few hydrocarbons have been detected in other goat cheeses [25,33]. It is worth noticing that none of the unsaturated hydrocarbons detected in Herreño cheese has been found in other types of goat cheese, except for phytene; the presence of this latter has been reported among the volatiles of other cheeses [11].

Although aromatic hydrocarbons come mainly from smoke, some of those detected in Herreño cheese like toluene, styrene and other alkylbenzene hydrocarbons (see Table 3) have also been detected in other unsmoked types of goat cheese [22,23,28].

As commented above, another characteristic of the volatile profile of the unsmoked Herreño cheese is the high number of terpenes and sesquiterpenes (see Table 3). Among these, α-pinene has been found in other goat cheeses [25], and the same can be said of limonene [22,23,24]. However, it must be noticed that the presence of so a high number of terpenes and sesquiterpenes as in this Herreño cheese had not been reported previously either in goat cheeses [22,24,29] or in other types of cheese [10,11], even though this type of components were also very numerous in La Serena cheese [34].

Concerning nitrogenated derivatives, it should be noticed that some of these components have been found in certain types of cheese, but not in goat cheeses; this is the case of benzonitrile [1] and of pyrazine and pyridine [35]. Finally, it only remains to add that some of the lactones and furan derivatives here detected have also been found in other types of goat cheese [25,26,36].

### 2.3. Comparison of the Smoked Herreño Cheese with Other Smoked Cheeses

The first issue to be taken into account when trying to compare the volatile profile of smoked Herreño cheese with other smoked cheeses is the scarce studies published on the volatile fraction of smoked cheeses in general and, particularly, of smoked cheeses made with goat milk. In fact, as far as we know, the only scientific works published about this latter type of cheese are those dealing with another type of Canary cheese, Palmero cheese, smoked with pine needles [12] and with dry prickly pear [15].

Although the fibre used for the above mentioned study of smoked Palmero cheese was not the same as in this work, some similarities have been found between the volatile profile of Palmero and smoked Herreño cheese. Thus, in both types of cheese, typical smoke components such as phenolic derivatives, cyclic and aromatic ketones, alkyl-aryl ethers and furan derivatives are among the most numerous and/or abundant components. Moreover, as in the smoked Palmero cheeses studied previously in our laboratory [12,15], the most abundant phenolic compounds in the smoked Herreño cheese are guaiacol, phenol and their derivatives. However, it must be noticed that among the aromatic ketones and diketones detected in smoked Herreño cheese, neither the methylindanones nor the cyclopent-2-en-1,4-dione had been detected in any of the Palmero cheeses studied previously or in other types of smoked cheese.

Although to a lesser extent than in the case of the above mentioned groups of components, the smoking process also enriches the volatile profile of Herreño cheese in esters, lactones, aldehydes, aromatic hydrocarbons and nitrogenated derivatives; these types of compounds were also present in smoked Palmero cheese [12,15], and in Oscypek, a type of smoked Polish ewe cheese [37].

In relation to esters, it is worth remembering that most of the esters coming from the smoking process of Herreño cheeses were methyl esters, and something similar was observed in Palmero cheese [12,15] and in Oscypek cheese [37]. Thus, the presence of methyl esters seems to be a characteristic of smoked cheeses since, in unsmoked cheeses, in general, ethyl esters are the predominant ones [8,22,23].

With regard to aldehydes, it is worth noticing that some of the aromatic aldehydes detected in smoked Herreño cheese, such as benzaldehyde, benzeneacetaldehyde or vanillin, were found in the headspace of smoked Palmero cheese [12,15]. On the other hand, crotonaldehyde (2-butenal), the most abundant aldehyde in smoked Herreño cheese (see Table 2) was also detected in Oscypek cheese [37].

Some of the nitrogenated derivatives present in the headspace of smoked Herreño cheese, such as those of nitrile (see Table 4), were also found in smoked Palmero cheese [12,15]; however, none of the pyridine and pyrazine derivatives detected in smoked Herreño cheese were found in Palmero cheese or in other types of smoked cheese. 

Finally, the smoking process provides Herreño cheese with a few alcohols, aliphatic hydrocarbons and terpenes. Concerning aliphatic hydrocarbons, it is worth noticing that, 4-methyl-2-pentene, one of the most abundant in smoked Herreño cheese, was not present in any of the Palmero cheeses studied previously [12,15]. Lastly, none of the four terpenes detected exclusively in the smoked Herreño cheese (see Table 3) was found in the headspace of Palmero cheese smoked with pine needles, despite terpenes were also quite abundant in this latter [12].

Taking into account that some of the components detected in the smoked Herreño cheese were not found in Palmero cheese smoked with dry prickly pear, which is one of the two smoking materials used for Herreño cheese, it could be thought that their presence in this cheese is due to the other vegetable matter used to produce the smoke: the fig tree wood.

### 2.4. Influence of the Position in the Smokehouse on the Volatile Profile of Smoked Herreño Cheese

As Table 1, Table 2, Table 3, Table 4 and Table 5 show, the statistical treatment of the data does not reveal, in general, significant differences between the abundance of most of the volatile components in the cheeses smoked at A and B positions. However the observation of a similar tendency in the components of the same group of volatiles could lead one to suppose some influence of the position on the concentration of some volatiles. This could be the case of acids (Table 1), whose area counts are somewhat higher in cheeses smoked at B position than at A, and of phenolic derivatives (Table 5), which exhibit slightly higher abundances in cheeses A, revealing a slightly higher smoking degree in these latter. This inverse relationship between the abundance of acids and of phenolic compounds in cheeses A and B seems to be in accordance with the lower acid abundance observed in smoked cheeses in relation to the unsmoked one. 

## 3. Material and Methods

### 3.1. Cheeses and Smoking Process

The cheeses subject of study were eight smoked Herreño cheeses and another unsmoked one. All the cheeses were manufactured at the same time, with milk from the same batch that, as commented above, was a mixture of goat, cow and sheep. The proportion of each type of milk in the total volume was approximately 75% for goat, 15% for sheep and 10% for cow. The manufacturing process, which has been described in detail elsewhere [38], was carried out in a local factory, following the traditional methods of production of this type of cheese. 

The smoked cheeses were subjected to the smoking process one day after their production process was initiated. All of them were smoked at the same time, in a smokehouse with external smoke generation. The smokehouse (see Figure 2) consists of a small-sized room with two holes: one for the smoke entry in the lower part of the middle of one wall and another for the smoke exit (chimney) in the ceiling, close to the middle of the opposite wall. During the smoking process, the smoke was continuously being generated and expelled to the atmosphere through the chimney.

For the smoking of the cheeses, they were placed on metal grilles at two different heights (1.20 m and 1.80 m from the floor), both of them above the smoke entry hole. The cheeses placed on the upper part were called A and those on the lower part B. Considering that smoke tends to go up, it was thought that A cheeses might be more exposed to the smoke action than B; for this reason, and with the aim of obtaining a similar smoking degree, B cheeses were kept in the chamber approximately 4 h and A cheeses only 2. The situation of the studied cheeses in the smokehouse can also be seen in Figure 2.

The smoke used was produced by the combustion of a mixture of fig tree wood (*Ficus carica*) and dry prickly pear (*Opuntia ficus indica*) in a combustion chamber next to the smokehouse. The distance between the smoke source and the smokehouse was approximately 1 m.

### 3.2. Generation of the Headspace and Extraction of its Components by SPME

The samples used for the study of the cheese volatiles were portions of the exterior of the cheeses (approximately 1 cm depth). This part was selected because the results obtained in previous studies carried out in our laboratory with other types of cheese revealed that this part was the richest in volatile components [12,15]. The samples were chopped and approximately 1 g of the chopped sample was weighed into a 4 mL amber vial screw top (acquired from Supelco, Bellefonte, PA, USA), sealed with a hole cap polytetrafluoroethylene/silicone septum, and stored frozen until its study.

Each vial containing 1 g of the cheese sample was introduced into a water bath maintained at 50 °C; then, the fiber was exposed to the headspace of the sample and was maintained for 60 min. The fiber used was coated with divinylbenzene/carboxen/polydimethylsiloxane (50/30 µm film thickness), and it was acquired from Supelco. The selection of the fiber type and the extraction conditions was based on previous studies carried out in our laboratory [11]. Each cheese was analysed in duplicate.

### 3.3. Study of the Extracted Compounds by Gas Chromatography/Mass Spectrometry

The extracted components were desorbed in the injector of a Hewlett-Packard gas chromatograph model HP 6890 Series II equipped with a mass selective detector 5973 and a Hewlett-Packard Vectra XM Series 4 computer. A fused silica capillary column was used (60 m length × 0.25 mm inside diameter × 0.25 µm film thickness; from Hewlett-Packard, Palo Alto, CA, USA), coated with a nonpolar stationary phase (HP-5MS, 5% phenylmethylsiloxane). The operation conditions were the same as in previous works [11,12,15].

The components were identified by their retention times and by comparison of their mass spectra either with those of standards or with spectra from a commercial library (Wiley 275.L, Mass Spectral Database, Rev. D.01.00, June 2000), as in previous studies [11,12,15].

Semi-quantification was based on arbitrary units of the base peak ion area counts divided by 10^5^.

The results given for the unsmoked cheese correspond to the average area counts of two different samples, whereas those given for the cheeses smoked at each position (A and B) are the average values of four cheeses; each of them was, in turn, analysed in duplicate.

### 3.4. Statistical Analysis

The statistical treatment of the data was carried out using the SPSS 19.0 software package (SSPS Inc., Chicago, IL, USA). One-way analysis of variance (ANOVA) was applied to the data to determine the presence of significant differences among volatile compounds of smoked and unsmoked cheeses (Tukey’s test, significant level *p* < 0.05).

## 4. Conclusions

Unsmoked Herreño cheese exhibits a very complex volatile profile, very rich in components, among which hydrocarbons, terpenes and sesquiterpenes deserve special attention due to their number, whereas acids and ketones are the most abundant. Although the unsmoked Hereño cheese has some volatile components that are common to other goat cheeses, such as acids, ketones and aldehydes, these are, in general, more numerous in Herreño cheese. In contrast, this cheese is poor in some groups of volatiles that are important in the aroma of other goat cheeses, such as alcohols and esters. Lastly, it is worth noticing the presence of a high number of hydrocarbons, including some unsaturated ones, of terpenes and sesquiterpenes and of nitrogenated derivatives, which can be considered characteristic of the unsmoked Herreño cheese, since they are very scarce or practically absent in other types of goat cheese.

Even more complex is the volatile profile of the smoked Herreño cheese, since it is enriched in other components coming from the smoking process, many of which are not present in the unsmoked one, such as phenolic derivatives and many ethers, ketones and diketones. On the other hand, the use of fig tree wood in combination with dry prickly pear for the smoking of Herreño cheese provides the smoked variety with specific components, among which special attention should be paid to pyridine and pyrazine derivatives due to their high number, and to others not detected previously in other types of smoked cheese, such as the methylindanones, the diketone cyclopent-2-en-1,4-dione, the unsaturated hydrocarbon 4-methyl-2-pentene and the four terpenes alloocimene, α-terpinolene, α-terpinene and β-camphor.

The high number of terpenes and sesquiterpenes both in unsmoked and smoked Herreño cheeses, which constitutes a distinguishing feature of this cheese, could be attributed to a great extent to the diet of the animals that produce the milk used for cheese production, based principally on autochthonous vegetation. Moreover, the presence of certain compounds from the characteristic flora of El Hierro Island, such as β-nicotyrine, could contribute to define markers specific for this type of cheese.

Therefore, it could be said that the milk used in the manufacture of the cheese, together with the smoking process performed on the island of El Hierro, results in two different varieties of Herreño cheese, each with well-defined characteristic volatile profiles that could be distinguished from those of other types of cheese.

Lastly, the position of the Herreño cheeses in the smokehouse does not seem to have a great influence on their volatile profile, given that statistically significant differences have not been found between cheeses A and B. However, the results obtained suggest a slightly higher smoking degree of the cheeses smoked at a greater height.
